# P-1607. Comparison of In-House Versus Reference Laboratory Antifungal Susceptibility Testing on De-Escalation Rates of Echinocandin Therapy for Candidemia

**DOI:** 10.1093/ofid/ofae631.1774

**Published:** 2025-01-29

**Authors:** Joseph Webb, Kristin Mondy, Theresa Jaso, Dusten T T Rose

**Affiliations:** Houston Methodist The Woodlands, Austin, Texas; Dell Seton Medical Center at the University of Texas, Dell Medical School at the University of Texas Austin, Austin, Texas; Ascension Seton Medical Center Austin, Austin, Texas; Dell Seton Medical Center at the University of Texas, Austin, Texas

## Abstract

**Background:**

The Mycoses Study Group Education and Research Consortium recommends healthcare facilities who routinely manage invasive fungal infections have access to “timely antifungal susceptibility testing,” and the Infectious Disease Society of America recommends antifungal de-escalation in candidemia within seven days to mitigate excessive drug costs and fungal resistance. In 2020, Ascension Seton hospital network converted from in-house (INH) antifungal susceptibility testing (AFST) to reference laboratory (RFL) testing.

**Figure:**
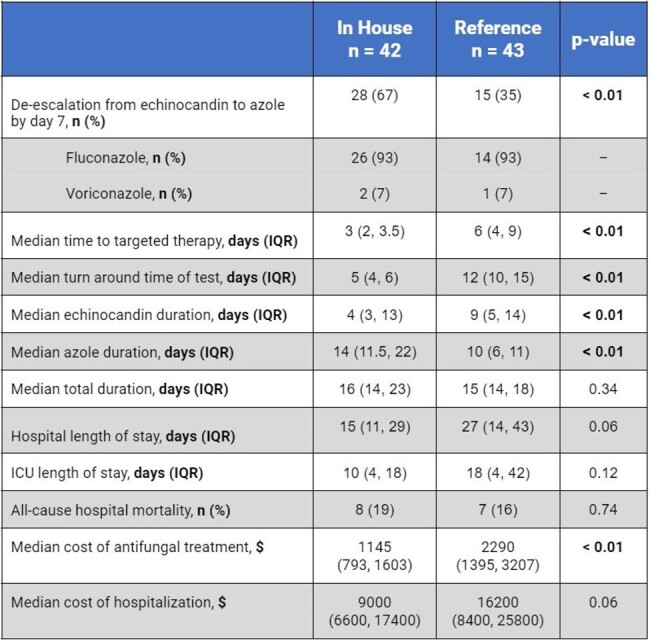

**Methods:**

This was a multicenter, retrospective cohort study evaluating the stewardship, clinical, and financial impact of RFL use for AFST in patients with candidemia. AFST was performed INH for isolates from May 2017 to Jul 2020 and by RFL for isolates from Aug 2020 to Sep 2023. The primary outcome was rate of antifungal de-escalation from echinocandin to azole therapy by day 7. Secondary outcomes include cost of antifungal treatment, hospital and intensive care unit (ICU) length of stay, days to targeted therapy, turn around time of test (TAT), and all-cause hospital mortality. Inclusion criteria were candidemia diagnosis and empiric echinocandin treatment. Exclusion criteria were diagnoses of candidemia with ocular, meningeal, or cardiac involvement; candidemia due to C. auris; or death before susceptibility resulted. Outcomes were analyzed by Wilcoxon rank sum.

**Figure:**
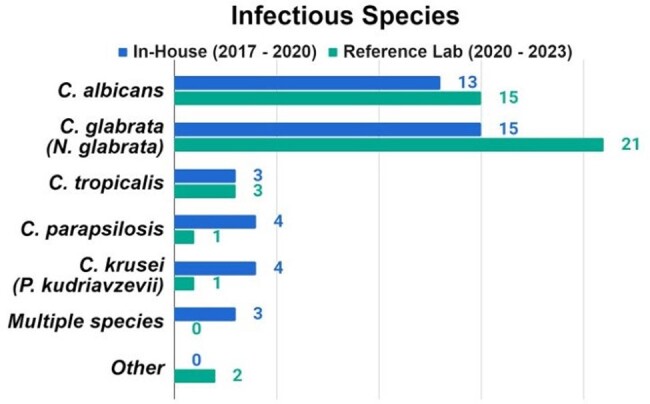

**Results:**

This study included 85 patients (42 INH and 43 RFL). The most common infectious species were *C. albicans* (31% INH vs 35% RFL) and *N. glabrata* (36% INH vs 49% RFL).Rate of de-escalation by day 7 was higher in the INH than RFL group(, 67 vs 35%, p< 0.01) . RFL use significantly increased days to targeted therapy (2 INH vs 6 RFL, p < 0.01), turn around days of test (5 INH vs 12 RFL, p < 0.01), echinocandin days (4 INH vs 9 RFL, p < 0.01), and cost of antifungal treatment ($1145 INH vs $2290 RFL, p < 0.01). There were no differences in additional secondary endpoints.

**Conclusion:**

Reference laboratory use for AFST was associated with significantly decreased echinocandin de-escalation rate, leading to longer turn around time of test, time to targeted therapy, and cost of antifungal treatment. In-house AFST offered faster susceptibility results and possibly cost savings.

**Disclosures:**

**All Authors**: No reported disclosures

